# Statewide Assessment of North Carolina Nurse Practitioners' Knowledge of and Attitudes Toward Suicide Awareness and Prevention: Protocol for a Statewide Mixed Methods Study

**DOI:** 10.2196/39675

**Published:** 2023-03-07

**Authors:** Charlene Whitaker-Brown, Judith Bacchus Cornelius, Jaleesa Smoot, Anjala Khadka, Arundhati Patil

**Affiliations:** 1 School of Nursing College of Health and Human Services University of North Carolina at Charlotte Charlotte, NC United States; 2 Department of Public Health College of Health and Human Services University of North Carolina at Charlotte Charlotte, NC United States; 3 William States Lee College of Engineering University of North Carolina at Charlotte Charlotte, NC United States

**Keywords:** mixed methods, suicide awareness, suicide prevention, nurse practitioner, knowledge and skills, stigma, suicide, awareness, nurse, public health, treatment, prevention, metal health, mental illness, United States, skills

## Abstract

**Background:**

Suicide is a major public health problem, which affects people of all ages and ethnicities. Despite being preventable, the rates of suicide have steadily climbed (more than a third) over the past 2 decades.

**Objective:**

Nurse practitioners (NPs) must be responsible for recognizing suicide risk and providing appropriate treatment referrals in addition to having an important role in suicide prevention. The reasons why NPs may not pursue suicide prevention training are their lack of suicide awareness and prevention, limited experiences with suicidal patients, and the stigma associated with mental illness. Before we begin to address the gaps within suicide awareness and prevention skills, we need to first examine NPs’ knowledge of and attitudes (stigma) toward suicide prevention.

**Methods:**

This study will comprise a mixed methods approach. First, quantitative data will be collected using the *Suicide Knowledge and Skills Questionnaire* *and the Suicide Stigma Scale (Brief version)* questionnaire. An email will be sent to the NPs explaining the purpose of the study. If they consent, they will click on a link to access the surveys on a secure site. In our previous research with this sample, email reminders to nonresponders after 2 and 4 weeks were sent. The quantitative component will be used to inform the qualitative interviews of this study. *The Suicide Knowledge and Skills Questionnaire* is a 13-item questionnaire comprising 2 subscales: suicide knowledge and suicide skills. All questions are rated on a 5-point Likert scale (1=completely disagree to 5=completely agree). The survey has been shown to differentiate between those with suicide training and those without and has a Cronbach α score of .84. *The Suicide Stigma Scale* (*Brief version*) is a 16-item survey that assesses stigma regarding suicide. The items are measured on a 5-point Likert scale (1: strongly disagree to 5: strongly agree) and have a Cronbach α of .98.

**Results:**

This study was funded by the Faculty Research Grants program through the Office of the Vice Chancellor for Research and Economic Development at the University of North Carolina at Charlotte. Institutional review board approval was obtained in April 2022. Recruitment occurred between summer and winter 2022. Interview conduction began in December 2022 and will conclude in March 2023. Data will be analyzed during spring and summer 2023.

**Conclusions:**

The study results will add to the literature on NPs’ knowledge of and attitudes (stigma) toward suicide prevention. It represents a first step in addressing gaps within suicide awareness and prevention skills, among NPs in their respective practice settings.

**International Registered Report Identifier (IRRID):**

PRR1-10.2196/39675

## Introduction

### Background

Suicide is a major public health problem, which affects people of all ages and ethnicities [1-6]. It is a multifactorial phenomenon often influenced by events that destabilize some of the pillars of daily life, such as family bonds, job satisfaction, economic stability, recreational life, and well-being [7]. Suicide is a global public health concern costing the lives of over 800,000 people per year [8]. It is among the 3 leading causes of death in those aged 15-44 years and the second leading cause of death among children and young people aged 15-29 years [8]. Among the strongest risk factors for suicide are a history of suicide attempts, mental illness, and self-harm [8,9]. Despite being preventable, the rates of suicide have steadily climbed (more than a third) over the past 2 decades [1-6]. Between 2007 and 2017, past-year suicidal ideation among college students nearly doubled (from 5.8% to 10.8%). Based on the Household Pulse Survey by the Centers for Disease Control and Prevention conducted from February 17 to March 1, 2021, 42.2% of participants aged 18-29 years reported indicators of depression in the past week [10]. The number of people who think about or attempt suicide is even higher [1-6]. The impact of COVID-19 with social distancing and isolation has exacerbated the risk of “deaths by despair” (suicide and substance use) during the pandemic [11,12]. Studies show that the pandemic has further widened the mental health treatment gap, and outpatient mental health services have been particularly disrupted [13]. Since the COVID-19 pandemic, vulnerable people (eg, those with a history of preexisting psychiatric disorders, low-resilience people, individuals who reside in high COVID-19 areas, those who have a family member to die of COVID-19, and health care providers) have experienced more suicide ideations post pandemic than before the pandemic [14-16]. Among those who are vulnerable and at risk of suicide because of COVID-19 are also those who are more fragile to the COVID-19-related economic crisis, analogous to the 2008 economic crisis [17]. In addition, evidence suggest that various factors including stress-related disorders of depression, posttraumatic stress disorder, and sleep disorders are associated with suicidal ideation, suicide attempts, and death by suicide [18]. Several factors have been found to contribute to suicidal ideation and behavior, including the presence of economic stressors, increased consumption of addictive substances (drugs and alcohol), domestic violence, intense exposure to anecdotes of pessimism and helplessness, and persistent feelings of entrapment, isolation, and loneliness. These factors can interact and have a synergistic effect, thus creating a vicious circle [19,20].

Moreover, many recovering patients with COVID-19 or those in the postacute phase experience psychosocial difficulties and such as loss of employment and financial distress. They also experience physical symptoms for an extended period [18]. Studies have highlighted the role of physical illness, especially among the oldest patients. Physical illness exerted a stronger motivational effect for suicide in old-old (≥75 years) attempters compared to their young-old (65-74 years) and middle-aged (50-64 years) counterparts. One-third of those who are >70 years of age, who had attempted suicide, attributed their act to somatic distress. Among those who had died by suicide, a greater incidence of physical illness was reported in the old-old compared to the young-old and middle-aged adults [19,20]. Most people who died by suicide had contact with the health care system in the year before their death [19,20]. It is documented that among people who died by suicide and who attempted to commit suicide, 83% of them had visited a primary care clinician, like a medical doctor (MD), nurse practitioner (NP), or physician assistant (PA) in the prior year, whereas 50% had visited a primary care clinician rather than a psychiatrist within 30 days of their death [21]. Moreover, older individuals who attend the emergency department (ED) with a physical illness are vulnerable individuals. The ED visit often represents a “sentinel event” that may signal a medical or psychosocial fracture in their established equilibrium. In addition, the ED represents a clinical setting where the elderly with both physical illness and greater suicidality risk frequently converge [19,20]. These missed opportunities are considered significant since risk and prevention factors are being overlooked. NPs serve a vital role in today’s health care system as primary care clinicians. In this role, NPs should be able to identify those at risk for suicide and intervene with prevention tactics and safety measures [22]. NPs of all specialties including family, pediatric, adult gerontology acute care, obstetrics/women’s health, and psychiatry should possess effective suicide assessment skills; however, suicide prevention training in schools of nursing is inadequate and limited to a few lectures [22]. Smith et al [23] have identified that knowledge about suicidal behavior and its assessment and treatment among clinicians has lagged in research in the health care. They have identified that gaps between research and practice could be indicative to the lack of appropriate training [23]. These gaps within suicide prevention knowledge are reflective of the need to increase suicide knowledge and awareness among primary care clinicians like NPs [24]. Health care clinician training is a top research priority identified by the National Action Alliance for Suicide Prevention [24].

In North Carolina, suicide is the third leading cause of death for children and young adults aged 10-34 years, the fourth cause of death in adults aged 35 to 54 years, ninth leading cause of death for those aged 55-64 years, and 17th leading cause of death for those aged 65 years and older [25]. On average, 1 person dies by suicide every 6 hours in the state [25]. The 2012 North Carolina Suicide Prevention Plan *encourages* but does not *mandate* suicide prevention training for health care professionals [26]. NPs must be responsible for recognizing suicide risk and providing appropriate treatment referrals, in addition to having an important role in suicide prevention [22]. One study suggests that NPs use more varied approaches in assessing, treating, and referring their geriatric patients with mental health problems [27]. This study also identified that NPs were more likely than their MD counterparts to express a lack of training and referral resources as barriers to treating symptoms such as depression among older adults [27]. The reasons why NPs may not pursue suicide prevention training is their lack of suicide awareness and prevention, limited experiences with suicidal patients, and stigma associated with mental illness [19,20,22]. Diamond et al [27] identified that even when funding is available to provide free training, behavioral change is gradual. Before we can begin to address gaps within suicide awareness and prevention skills, we need to first examine NPs’ knowledge of and attitudes (stigma) toward suicide prevention.

### Aims

Our long-term research goal is to increase suicide knowledge and awareness among NPs in the state of North Carolina. The purpose of this study is to use the Theory of Nursing Care of Patients at Risk of Suicide [28] as a guiding framework to identify NPs’ suicide awareness, attitudes (confidence, stigma, facilitators, and barriers), knowledge, and prevention skills. Knowledge from this study is essential to inform the development of policy and a targeted intervention to increase NPs’ knowledge of and improve their attitudes toward suicide awareness and prevention.

The aims of this study are to (1) explore NPs’ knowledge of and attitudes toward suicide awareness and prevention; (2) examine the differences in NPs’ suicide awareness and prevention skills based on practice specialty; (3) identify enablers and barriers to NPs’ awareness and confidence of suicide prevention skills.

### Theoretical and Conceptual Framework

Billings [29] identified that nurses should be prepared to recognize suicide risk factors and risk predictors so that they can make sensible and sound nursing decisions. Midence et al [30] used a questionnaire to investigate a sample of nurses’ (n=27) views on the effects on them of patient suicides, and 45% (n=12) of the respondents said that suicide could be prevented if all patients were assessed for suicide risk. For this study, the Theory of Nursing Care of Patients at Risk for Suicide and the Theory of Planned Behavior were used in developing the study aims, design, measure selection, and qualitative inquiry and analysis. Specifically, the Theory of Nursing Care of Patients at Risk of Suicide was used as a guiding framework to identify NPs’ suicide awareness, attitudes (confidence, stigma, facilitators, and barriers), knowledge, and prevention skills [28]. Based on a grounded theory approach, the Theory of Nursing Care of Patients at Risk of Suicide guides the nurses as they initiate and maintain therapeutic relationships with patients who are at risk for suicide [28]. The core category of this theory is the provision of safe and compassionate care via the therapeutic relationship. Additional categories linked to this core category include the following: providing holistic assessments, providing protection, providing basic care, and promoting healing through advanced care [28]. This theory has been identified as one that can advance the quality of care provided by nurses with their potential to instill hope in patients that have lost their ability to cope with life’s events [28]. Moreover, the theory of planned behavior posits that human behavior is guided by three considerations leading to the formation of intention and then behavior: (1) beliefs about likely outcomes and evaluations of behaviors (*behavioral beliefs*), (2) beliefs about normative expectations of others and motivation to comply (*normative beliefs*), and (3) beliefs about the presence and power of factors that may facilitate or impede the performance of the behavior (*control beliefs*) [23,31]. Increasing awareness of suicide-related facts, risk factors, and prevention approaches may increase knowledge and, in turn, confidence in skills related to suicide prevention via influencing behavioral, normative, and control beliefs. Thus, increases in knowledge and confidence in skills may make it more likely that an individual will engage in suicide prevention behaviors such as suicide risk assessments and referrals [23].

## Methods

### Study Design

An observational mixed methods approach will be used for this study. Because the data collection questionnaires include forced choices and the prevention needs of NPs based on specialty practice have not been explored, the mixed method design is appropriate to address the objectives of the study. Descriptive statistics will be used to compute means and SDs for numerical variables and frequencies for categorical variables. Demographic and background information regarding age, gender, professional role, education, and previous suicide prevention training will be collected [32].

#### Qualitative Data

A cross-sectional survey design including a series of interviews for a small selection of nurses who have completed the survey will be used. Qualitative interviews will be audio recorded and fully transcribed removing any identifiable data to preserve participant anonymity [8]. Qualitative content analysis will be conducted on the open-ended questions [33]. We will use interpretative phenomenological analysis (IPA). IPA makes sense of the participant’s experiences by seeking to understand the cognitive, affective, linguistic, and physical being while maintaining the researcher as an integral part of the sense-making process [34-38]. This methodology is best used in studies where the objective is to explore the meaning behind the experiences of participants. IPA is an especially fitting research approach when the researcher intends on asking complex, broad, and open-ended questions [34-38], as is the case in this research proposal. In keeping with the open-ended nature of qualitative research, this method allows the researcher to explore the research question in a flexible nonprescriptive manner, thus facilitating a more thorough exploration [34-38].

#### Quantitative Data

First, quantitative data will be collected using the *Suicide Knowledge and Skills Questionnaire* [39] *and the Suicide Stigma Scale (Brief version)* [40] questionnaire. An email will be sent to the NPs explaining the purpose of the study. If they consent, they will click on a link to access the surveys on a secure site. In our previous research with this sample, email reminders to nonresponders after 2 and 4 weeks were sent [41]. The quantitative component will be used to inform the qualitative interviews of this study.

#### Quantitative Component-Measures

The Suicide Knowledge and Skills Questionnaire [39] is a 13-item questionnaire comprising 2 subscales: suicide knowledge and suicide skills. The items in this survey were based on the 100-item Suicide Opinions Questionnaire (SOQ) [23,39]. The SOQ consists of 8 subscales derived using expert consensus and empirical tests for internal consistency. The Suicide Knowledge and Skills Questionnaire has 9 items that parallel the items on the SOQ, and it will assess participants’ knowledge about suicidal behavior and comfort dealing with suicidal clients [39]. The Suicide Knowledge subscale included 9 items that will reflect either truth or falsehood about suicide. Moreover, the Suicide Skills subscale has 4 items that assess the respondents’ comfort dealing with suicidal clients in terms of their confidence in their training, skills, and supervision received [23,39]. All questions are rated on a 5-point Likert scale (1 completely disagree, 2 disagree, 3 don’t know, 4 agree, and 5 completely agree). An example item is as follows: “I am comfortable asking direct and open questions about suicide,” “I have received the training I need to engage and assist those with suicidal desire and/or intent [23,39].” The questionnaire takes 10 minutes to complete. The survey has been shown to differentiate between those with suicide training and those without and has a Cronbach α score of .84 [23,39]. *The Suicide Stigma Scale (Brief version)* [40] is a 16-item survey that assesses stigma regarding suicide. The items are measured on a 5-point Likert scale (1, strongly disagree to 5, strongly agree), has a Cronbach α of .98 and takes 10 minutes to complete [40].

#### Demographic Survey

Participants will report demographic data such as age, gender, specialty, education level, years of NP experience, previous suicide prevention training, and experience with suicidal patients.

### Recruitment and Participants

#### Sampling Strategy and Power Analysis

The researchers will use the North Carolina Board of Nursing database for NPs registered to practice in the state. Inclusion criteria are as follows: (1) a licensed NP in the state of North Carolina and (2) able to give consent. Exclusion criteria include not being able to provide consent and not being a NP in the state of North Carolina. Our study is powered to detect the primary outcome of suicide knowledge subscale differences among nursing specialties and controlling for other demographic characteristics. Smith et al [23] used an analysis of covariance model (ANCOVA) design and reported between-group differences of approximately 0.2 and an interrespondent SD of 1.53, which leads to an implied effect size of f=0.13. However, since their study involves comparisons among other health professionals as well, we account for effect size uncertainty by using a mid-point between this informative choice and an uninformative small effect size of f=0.10, leading to f=0.115 [23]. We assume an attrition level in line with a recent study in which we used the same recruitment strategy and population [41]. We anticipate sufficient respondents from (or grouping respondents into) 5 core specialties (family, pediatric, adult gerontology acute care, obstetrics/women’s health, and psychiatry). With an 80% power and a 5% alpha, the required sample size before attrition to detect group differences of f=0.115 among 5 specialties, with a conservative upper bound of 0.25 for the multiple correlation from demographic confounders, is 5,535 [42]. This is smaller than the population accessible by the researchers via email (N=10,916). In our previous study, we were able to obtain over 2000 responses without incentives; in this study, we will give US $10 gift cards (first 150 participants) [41].

### Study Setting

First, quantitative data will be collected using survey data via a questionnaire. An email will be sent to the NPs, and upon consent, they will have to access the surveys on a secure site. The quantitative component will utilize semistructured interviews via Zoom (Zoom Video Communications, Inc). An interview guide will be developed guided by the Theory of Nursing Care of Patients at Risk of Suicide [28] and quantitative data responses (Multimedia Appendix 1). Consistent with qualitative interviewing methods, we will begin the interview with an introductory statement: “Tell me about your previous experiences with suicide.” The process will continue addressing questions focused on suicide knowledge (providing protection and basic care and promoting healing) and awareness (confidence, facilitators and barriers, and experiences). Participants will receive a US $50 gift card for completing the interview process.

### Data Collection, Management, and Analysis

Demographic data will be analyzed using descriptive statistics. Differences in questionnaire responses across specialists while controlling for other demographic characteristics will be analyzed with ANCOVA. These data will be analyzed using Statistics for Macintosh (version 28, IBM Corp).

Our qualitative data sampling strategy will use NPs who complete the quantitative surveys. NPs will be able to indicate if they are interested in participating in a virtual interview lasting about 50 minutes to identify facilitators and barriers to suicide knowledge and awareness. A representative sample of NPs based age, gender, ethnicity, specialty practice, and location (rural vs urban) using an address to identity (N=15 or until saturation) will be interviewed for part two of the study.

The semistructured interviews will be recorded and transcribed verbatim. The data will be managed using NVivo software (QSR International) for content analysis and analyzed for points of convergence and divergence.

### Confidentiality

Participants will be informed that safeguards have been put in place to protect confidentiality and anonymity. All study-related information and spreadsheets will be stored in a locked office and file cabinet. All participant information will be coded by identification number to maintain confidentiality. We will protect confidentiality by removing all identifiers. Only members of the research team will have access to the data, and only aggregate data will be presented for publication.

### Ethics Approval

Approval from the University of North Carolina at Charlotte institutional review board (IRB-22-0925) was required since the protocol is categorized as a research study [43]. The study’s protocol, surveys, and informed consent forms were reviewed to ensure respect, fairness, and safety in human subjects research [44]. The protocol was followed in accordance with the standards for human subjects research. The study participants will be given the opportunity to opt out and will be informed of their right to privacy. Each member of the research team has completed the required training on proper methods of conducting research in compliance with federal and state requirements [44].

### Instruments

The instruments and the reliability and validity values to be used in our pilot study are shown in Table 1.

**Table 1:** Instruments and reliability values of this study.

Study outcome	Instrument	Reliability values
Demographic survey: participants will report demographic data such as age, gender, specialty, education level, years of nurse practitioner experience, previous suicide prevention training, and experience with suicidal patients.	Demographic survey	N/A^a^
Nurse practitioner knowledge and skills-Suicide	Suicide Knowledge and Skills Questionnaire [39]	Cronbach α score of .84
Nurse practitioner stigma-suicide	Suicide Stigma Scale (Brief version) [40]	Cronbach α score of .98

^a^N/A: not available.

### Timeframe

The project can be completed within 12 months. We anticipate no problems in recruiting subjects for the proposed study. The timeline for this study is shown in Table 2.

**Table 2:** Research tasks timeline.

Research tasks	Jan-Apr 2022	May-Dec 2022	Dec-Feb 2023	Feb-May 2023
Obtain institutional review board approval, send surveys, and email reminders	✓	—^a^	—	—
Recruit for qualitative interviews and analyze quantitative data	—	✓	—	—
Conduct interviews and analyze data	—	—	✓	—
Interpret and compare data and disseminate findings	—	—	—	✓
Apply for funding	—	—	—	✓

^a^Not applicable.

### Strengths and Limitations

In this pilot feasibility study, we will have a sample of convenience, which may result in response bias. We are recruiting participants who are NPs in the state of North Carolina. Hence, the findings may be different from those who are MDs or PAs. For future research, we may consider the inclusion of additional clinicians like MDs or PAs. The strengths include the capacity to reach all registered NPs in the state of North Carolina.

## Results

### Overview

The institutional review board’s approval was obtained in April 2022. Recruitment occurred between summer and winter 2022. Data analysis is to be completed by the second quarter of 2023. Our study is expected to conclude in the second quarter of 2023. We will submit a manuscript for publication consideration by the third quarter of 2023.

### Anticipated Findings

We hypothesize that NPs will endorse self-efficacy working with suicidal clients, familiarity using evidence-based assessment procedures, and identifying best practices for suicide prevention [45].

## Discussion

### Principal Findings

In this study, we describe a protocol for examining NPs’ knowledge of and attitudes (stigma) toward suicide prevention. This represents a first step in addressing gaps within suicide awareness and prevention skills, among NPs in their respective practice settings or sites. Telepsychiatry interventions and digital tools (eg, mobile apps, internet chatbots, and videoconferencing) have proliferated rapidly in response to the COVID-19 emergency [10]. It is important for NPs to be aware of the various resources to help them with their assessment of patients. One of the most effective ways to combat serious public health problems, like suicide, is to increase knowledge about risk factors, assessment procedures, and treatment options [23]. Gold-standard evidence-based training approaches aim to improve both knowledge and skills about suicide and how to intervene and include some form of didactic training (in-person or web-based) with a demonstration or modeling of suicide prevention skills, opportunities to practice these skills, and expert coaching and feedback on practicing these skills [21]. Many also target attitudes for increasing willingness and motivation to engage in suicide prevention activities given that stigma, anxiety, and unhelpful myths about suicide are common among the general population and professionals [21]. Lastly, we hope this protocol paper will provide a roadmap and resources for other researchers who would like to implement a similar approach in their studies [45]. It has been suggested that when screening programs and training programs have been implemented in different medical care settings patients at risk for suicide who would otherwise have gone unnoticed have been identified [21].

### Future Directions

We have developed a strategic dissemination plan. The research process and our findings will be shared with university colleagues and clinical providers, in a peer-reviewed journal, and at a research conference.

### Conclusions

This study will close the gap in identifying NPs’ knowledge of and attitudes toward suicide awareness and prevention. In addition, the results with help to examine differences in NPs’ suicide awareness and prevention skills based on practice specialty. Finally, we expect our results to identify enablers and barriers to NPs’ awareness and confidence in suicide prevention skills. The study’s findings will add to the current literature on NPs’ knowledge of and attitudes (stigma) toward suicide prevention. Our study results may inform practice, policy, further research, and training. The results may shape how members of the health care system respond to people who are at risk of suicide and their families [36]. It represents a first step in addressing gaps within suicide awareness and prevention skills, among NPs in their respective practice settings.

## Appendix

Multimedia Appendix 1 Interview guide.

## Abbreviations

ANCOVA: analysis of covariance model
ED: emergency department
IPA: interpretative phenomenological analysis
MD: medical doctor
NP: nurse practitioner
PA: physician assistant
SOQ: Suicide Opinions Questionnaire

## Data Availability

The data sets generated during or analyzed during this study are not publicly available but are available from the corresponding author on reasonable request.
