# Supplementary material for: Statewide Assessment of North Carolina Nurse Practitioners' Knowledge of and Attitudes Toward Suicide Awareness and Prevention: Protocol for a Statewide Mixed Methods Study
Source: JMIR Res Protoc. 2023 Mar 7;12:e39675. doi: 10.2196/39675 (PMC10031437; doi:10.2196/39675)
Supplement: Multimedia Appendix 1 [file resprot_v12i1e39675_app1.docx]

1. What type of nurse practitioner are you?
2. What type of setting do you provide patient care?
3. Tell me about your previous experiences with suicide.
4. Tell me about your knowledge related to suicide.
   1. For example, can you provide information about the rate of suicide?
5. On a scale of 1-10 with 10 being most confident and 1 being least confident, what is your confidence level assessing patients for suicide.
6. Identify any barriers that affect your confidence assessing patients and asking them questions related to suicide.
7. Identify what you need as an advanced practice nurse (resources) to help facilitate an open nonbiased discussion with your patient(s) related to suicidal thoughts and ideations.
8. The importance of carrying out a holistic assessment of patients’ needs is paramount. What risk factors have you identified in patients that can indicate increased suicide risk and or ideation?
9. It is essential for nurses to provide protection in all settings including the clinic, hospital and within the therapeutic relationship, as well as basic and advanced nursing care.
   1. What are your thoughts related to providing protection for those at risk for suicide that need basic care?
   2. How would you help promote health and assure basic care and needs are met? Give at least 1-2 examples.
10. In offering advanced nursing care, nurses merge both the art and science of nursing to include empathy, compassion and facilitating degrees of hope for the future based on their accurate assessment of patients’ needs and aspirations. Identify several assessment skills needed and or questions that you think are important to ask questions to identify their risk for suicide and need for help and supportive intervention.

Reference:

Sun FK, Long A, Boore J, Tsao LI. A theory for the nursing care of patients at risk of suicide. *J Adv Nurs*. 2006;53(6):680-690. doi:10.1111/j.1365-2648.2006.03774.x
